# Prognostic Impact of Blood Pressure Variability on Aortic Dissection Patients After Endovascular Therapy

**DOI:** 10.1097/MD.0000000000001591

**Published:** 2015-09-25

**Authors:** Lei Zhang, Wen Tian, Rui Feng, Chao Song, Zhiqing Zhao, Junmin Bao, Aijun Liu, Dingfeng Su, Jian Zhou, Zaiping Jing

**Affiliations:** From the Department of Vascular Surgery, Changhai Hospital, Second Military Medical University, Shanghai, China (LZ, WT, RF, CS, ZZ, JB, ZJ); Department of Pharmacology, Second Military Medical University, Shanghai, China (AL, DS); and Department of Surgery, Changhai Hospital, Second Military Medical University, Shanghai, China (JZ).

## Abstract

Hypertension has been deemed as a pivotal risk factor for the development of aortic dissection; however, the importance and prognostic significance of blood pressure variability (BPV) in aortic dissection are always ignored.

A total of 173 acute type B aortic dissection patients were enrolled in and retrospectively reviewed between January 2009 and November 2013. There were 74 patients with high BPV and 99 with low BPV stratified by preoperative mean BPV. Technical success was achieved in all patients. The proportions of hypertension and general anesthesia were significantly higher in the high BPV group (70.3% vs 55.6% and 77% vs 62.6%, *P* = 0.049 and 0.043, respectively). The risk of aorta-related death in the high BPV group was apparently higher than the low BPV group (28.4% vs 9.1%, *P* = 0.001). By performing multivariable logistic regression, we found history of hypertension was likely to be a risk factor of BPV (95% confidence interval [CI]: 1.010–3.911), and high BPV was an independent predictor of aorta-related death (95% CI: 1.671–9.587). The difference of aorta-related mortality was pronounced between high and low BPV subgroups regardless of the refractory hypertension (41.4% vs 14.3% and 20.0% vs 7.0%, *P* = 0.023 and 0.037, respectively). The thrombosis ratio of false lumen was significantly higher in the low BPV group at 3-month (72.4 ± 17.5% vs 51.8 ± 11.6%, *P* < 0.001) and 6-month (86.4 ± 9.1% vs 69.7 ± 7.9%, *P* < 0.001).

High BPV is an independent risk factor for the prognosis of aortic dissection. Further studies on BPV might provide new preventive and therapeutic strategies for aortic dissection.

## Abstract 2

Supplemental Digital Content is available in the text

## INTRODUCTION

Acute type B aortic dissection (ABAD) is a life-threatening medical emergency with in-hospital mortality as high as 10.7% treated medically and 31.4% treated surgically.^1^ Compared with traditional open surgery, thoracic endovascular aortic repair (TEVAR) has been chosen as a less invasive alternative for the treatment of complicated ABADs,^2–5^ especially for elder patients with greater comorbidities^6^ and anatomic variations of the aortic arch.^7^ However, postoperative adverse events such as aortic rupture, stroke, paraplegia, and retrograde type A aortic dissection have influenced the therapeutic effect of TEVAR.^8^ During the past decade, there has been an increasing interest in the predictor of outcomes in ABAD after TEVAR, and female gender, renal failure, and in-hospital hypotension have been reported as independent predictors.^9^ It is of great importance to identify a more stable and easier available predictor of outcomes for TEVAR. It is also conceivable that the prognosis of ABAD can be improved by regulating the predictor.

More than 70% aortic dissection patients have history of hypertension,^1^ and medical management centered on blood pressure control is accepted standard of care in the routine clinical work. But the importance and prognostic significance of blood pressure variability (BPV) are always ignored. Therefore, we considered whether BPV would influence the thrombosis process of false lumen and be a significant predictor of outcomes for TEVAR. In this study we evaluated the effect of BPV on the prognosis of ABAD and explored the possible mechanism.

## METHODS

The study protocol complied with the declaration of Helsinki and was approved by the ethics committee of our hospital. Written informed consent forms were obtained from each patient before intervention.

### Patient Population

A retrospective review of aortic dissection patients treated in our department was performed. The diagnosis of aortic dissection was confirmed by computed tomography angiography on a 64-row (0.6 mm)^10^ CT scanner (Siemens, Munich, Germany) in all patients. The inclusion criteria were as follows: (1) ABAD, not including intramural hematoma or penetrating atherosclerotic ulcer; (2) no congenital connective tissue diseases or traumatic dissections; (3) no previous open surgery or endovascular therapy for aortic diseases.

### Protocol for the TEVAR Procedure

All the interventions were performed in the digital subtraction angiography room. A standard percutaneous puncture of the access artery was performed, and heparin was given intra-arterially (80 U/kg). Angiography was routinely used to identify the true lumen and primary entry tear, followed by selective catheterization of the target vessel. A stiff wire was then placed, entering into the true lumen, following which the stent grafts were advanced and deployed consecutively to cover the primary entry tear. Five stent graft systems, ∼ 10 to 20% oversized, were used. If >1 graft was deployed, the overlap length was 30 to 40 mm. Cerebrospinal fluid drainage was used only when long-segment aortic coverage was planned. A vascular closure device was used to manage the access site after intervention. More details to the TEVAR procedure was presented in Table 1.

**TABLE 1:**
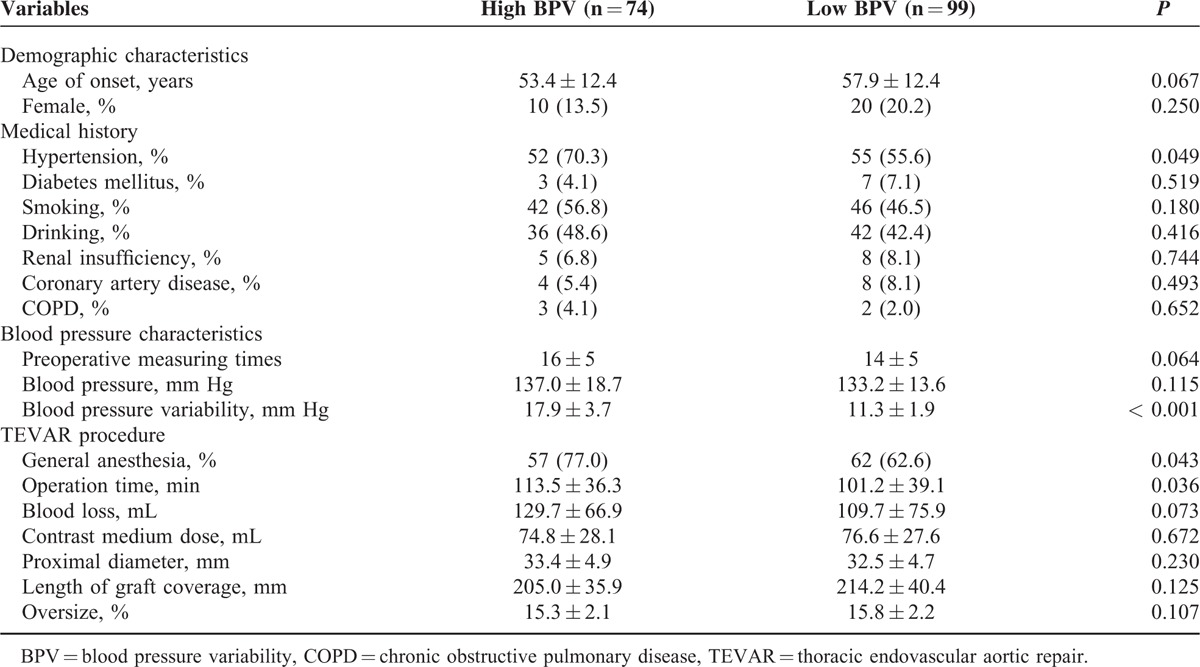
Demographic and Clinical Data of the Study Cohort

### Definitions

According to the Stanford classification, ABAD was all dissections that did not involve the ascending aorta, presenting within 2 weeks of symptom onset.^1,11,12^ Intramural hematoma was presented with regionally thickened aortic wall without obvious true lumen, false lumen, or intimal flap.^13^ Penetrating aortic ulcer was a deep ulcerated lesion in the wall of aorta.^14^ Refractory hypertension was uncontrolled systolic blood pressure despite treatment with >3 antihypertensive drugs from at least 3 different classes including a diuretic.^15^ Technical success was technically successful placement of stent graft at the intended target location.^16^ Mortality referred to the aorta-related death, which was defined as death from aortic rupture, malperfusion, or proximal dissection.^17^ Perioperative and postdischarge events included retrograde type A dissection, conversion to open surgery, rupture of iliac access artery, ancillary procedure, major stroke, and paraplegia.

### Measurements of Blood Pressure and Blood Pressure Variability

All hypertensive patients received antihypertensive agents via oral route. Perioperative blood pressure was measured and recorded every 4-hour during the hospitalization. Rigorous surveillance was applied for those patients with high blood pressure (systolic blood pressure ≥180 mm Hg). Until now, there was no criterion about how to quantify BPV in routine practice. Almost all previous BPV studies were based on systolic blood pressure.^18,19^ In the present study, BPV was defined as the standard deviation of systolic blood pressure.^20^ Patients were stratified into high and low BPV groups by mean BPV. The high BPV group was defined as those patients whose BPV was higher than mean BPV, and the low BPV group was defined as those patients whose BPV was lower than mean BPV.

### Computed Tomography Image Analysis

All patients who underwent the TEVAR procedure were regularly followed up at 3-, 6- and 12-month postoperatively, and then annually afterward until the end of this study in June 2014. Computed tomography angiography examinations were arranged at 3- and 6-month postoperatively. A centerline of flow was generated by using the semi-automated centerline algorithms on the dedicated three-dimensional workstation (Aquarius WS 3.7.0.13, TeraRecon Inc, San Mateo, CA).^10^ Once the whole aortic area was chosen, the aortic diameter was automatically generated in every different slice. Then the aortic maximum diameter obtained (see Figure S1, http://links.lww.com/MD/A423, Supplemental Content, which demonstrates measurement of aortic diameter in acute type B aortic dissection). The aortic maximum diameter decrease ratio at each follow-up point was calculated using the following formula: 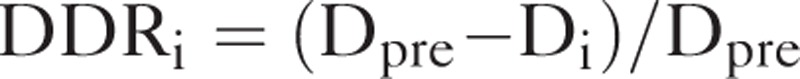


where D_pre_ and D_i_ stand for the aortic maximum diameter before TEVAR and at i months follow-up, respectively, and DDR_i_ stands for the aortic maximum diameter decrease ratio at i months follow-up.^21^

The areas of true lumen, contrast-enhanced (perfused) false lumen, and noncontrast-enhanced (thrombosed) false lumen were manually determined on the CT slices (see Figure S2, http://links.lww.com/MD/A423, Supplemental Content, which demonstrates the measurement of interested areas in pre- and post-TEVAR CT slices; see Figure S3, http://links.lww.com/MD/A423, Supplemental Content, which demonstrates measurement processes of the whole and thrombosed false lumen areas in high and low BPV groups at different follow-up points).^22^ The thrombosis ratio of false lumen was defined as the total thrombus volume over whole false lumen volume and was calculated according to the following formula: 
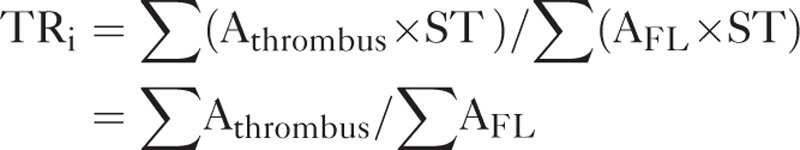


where TR_i_ stands for the thrombosis ratio at i months follow-up, A_thrombus_ and A_FL_ stand for the thrombus area and the whole false lumen area on the cross-sectional images, respectively, and ST stands for the slice thickness of the scan protocol.^21^

### Statistics Analysis

All analyses in the study were performed using IBM SPSS 19.0 (IBM Corp., Armonk, NY). Data were expressed as numbers, proportions, and mean ± SD. Chi-squared or Fisher's exact test was used to compare the categorical variables. Continuous variables were compared using two-group *t* test or the nonparametric Mann–Whitney test. Multivariable logistic analysis was performed to evaluate the prognostic values of demographical and therapeutic variables on type B aortic dissection. Odds ratios were given with 95% confidence intervals (CI). The null hypothesis was rejected for values of *P* < 0.05.

## RESULTS

**FIGURE 1:**
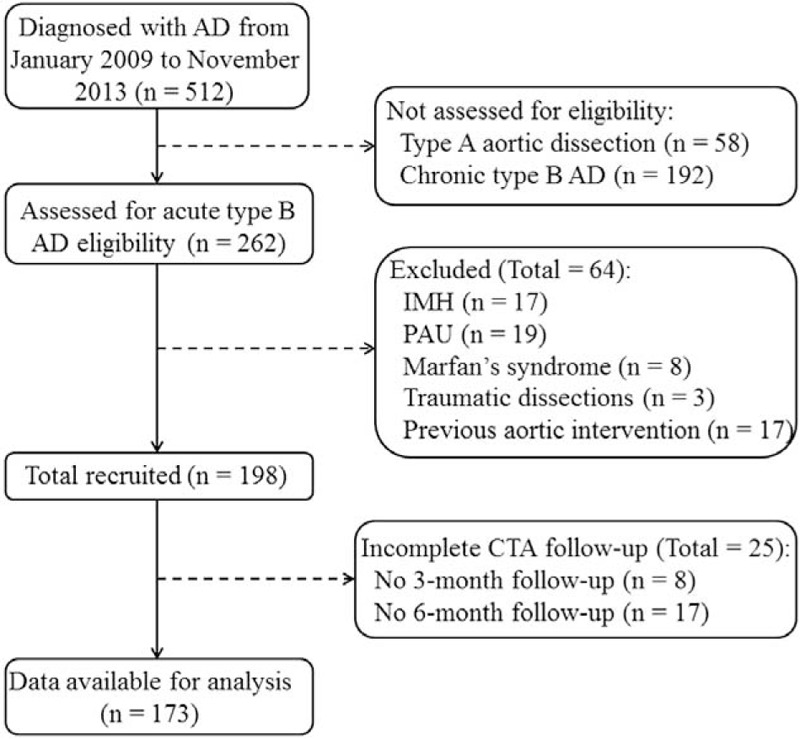
Flow diagram of the patients included in the study. AD = aortic dissection, CTA = computed tomography angiography, IMH = intramural hematoma, PAU = penetrating atherosclerotic ulcer.

From January 2009 to November 2013, a total of 512 consecutive aortic dissection patients were initially treated with TEVAR in our center, and 173 patients were enrolled in the study according to the inclusion criteria (Figure 1). Most of aortic dissections were complicated, and the indications for TEVAR were presented as following: 36 malperfusion; 41 rapid enlargement of aortic diameter; 24 persistent intractable chest/back pain; 26 impending rupture. Eleven patients had not only refractory chest/back pain but also malperfusion indications. The rest of 57 patients had refractory hypertension.

### Patients’ Characteristics

**FIGURE 2:**
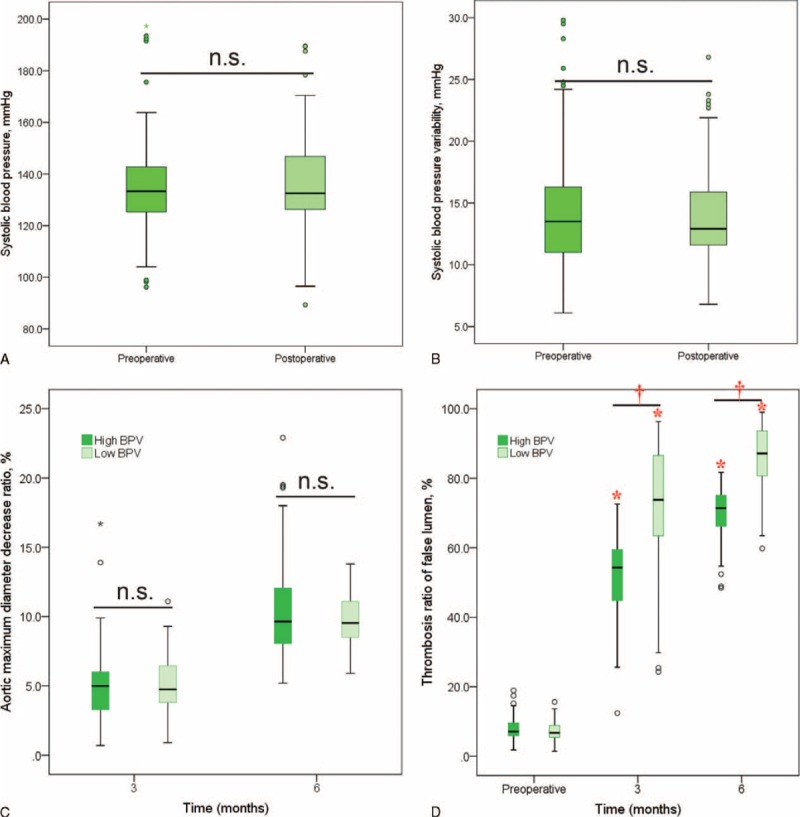
Measurement results of blood pressure, blood pressure variability, aortic maximum diameter decrease ratio, and thrombosis ratio of false lumen. (A and B) There was no significance of blood pressure and blood pressure variability between pre- and postoperative. (C) No significance of aortic maximum diameter decrease ratio was found between high and low BPV groups at 3- and 6-month follow-up points. (D) The thrombosis ratio of false lumen was significantly higher compared with preoperative, and statistical significance of thrombosis ratio was demonstrated between high and low BPV groups at 3- and 6-month follow-up points. n.s. indicates no significance. ^∗^Indicates *P* < 0.001 compared with preoperative; ^†^Indicates *P* < 0.001 when low BPV group compared with high BPV group.

No significant differences were observed in blood pressures (134.8 ± 16.0 mm Hg vs 135.8 ± 16.3 mm Hg, *P* = 0.588, Figure 2A) and BPVs (14.1 ± 4.3 mm Hg vs 13.6 ± 3.5 mm Hg, *P* = 0.206, Figure 2B) between pre- and postoperative groups. There were 74 patients (42.8%) with high BPV and the rest 99 patients (57.2%) with low BPV stratified by preoperative mean BPV.

Technical success was achieved in all patients. The ages of onset were 53.4 ± 12.4 years and 57.9 ± 12.4 years in high and low BPV groups, respectively. The proportions of hypertension and general anesthesia in the high BPV group were significantly higher than low BPV group (70.3% vs 55.6% and 77% vs 62.6%, *P* = 0.049 and 0.043, respectively). The operation time was apparently longer in high BPV group (113.5 ± 36.3 min vs 101.2 ± 39.1 min, *P* = 0.036). No differences of other characteristics were observed between the 2 groups (Table 1).

### Risk Factors of Blood Pressure Variability

Multivariable logistic regression was performed to explore the potential underlying relations between known factors and BPV in our study. The result suggested that history of hypertension was likely to be a risk factor of BPV (95% CI: 1.010–3.911, *P* = 0.047) (Table 2).

**TABLE 2:**
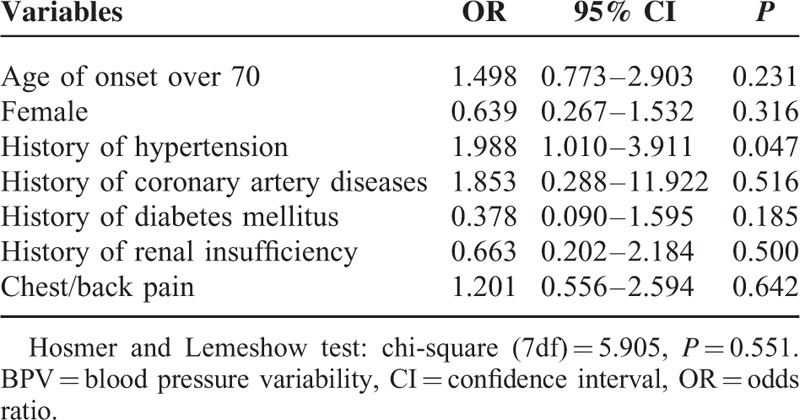
Multivariable Logistic Regression Model for Risk Factors of High BPV

### Blood Pressure Variability and Outcomes

**FIGURE 3:**
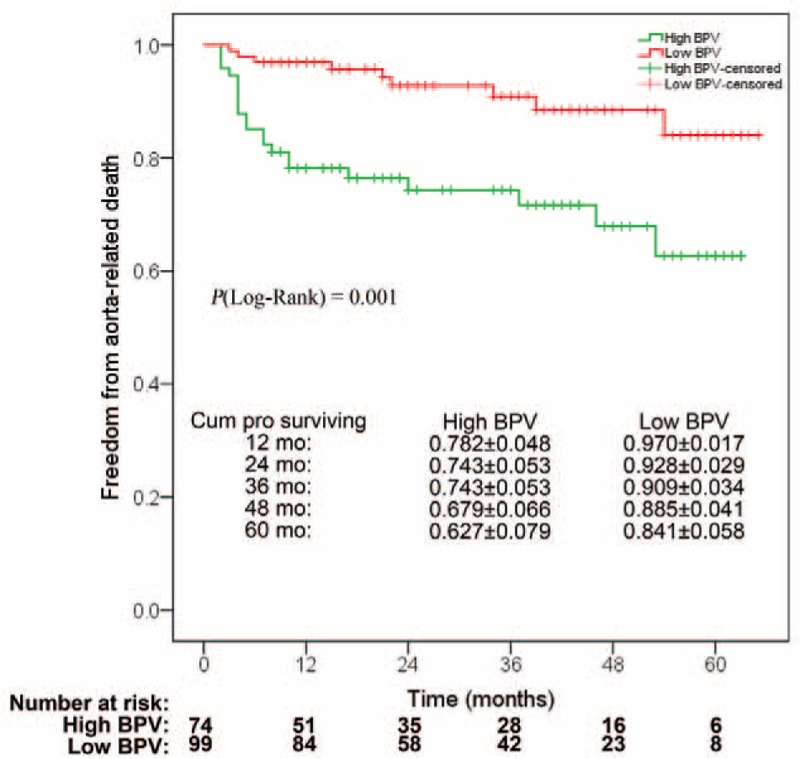
Kaplan–Meier estimates of cumulative proportion of freedom from aorta-related death in high and low BPV groups. The probabilities were presented as mean ± SEM.

The median length of follow-up was 25 (range, 2–65) months. The risk of aorta-related death was significant higher in the high BPV group (28.4% vs 9.1%, *P* = 0.001). No differences of perioperative and postdischarge adverse events were observed between the 2 groups (Table 3). The cumulative proportion of freedom from aorta-related death over the full follow-up period in high and low BPV groups was presented in Figure 3. The probability value of Log-rank test between the 2 curves was 0.001.

**TABLE 3:**
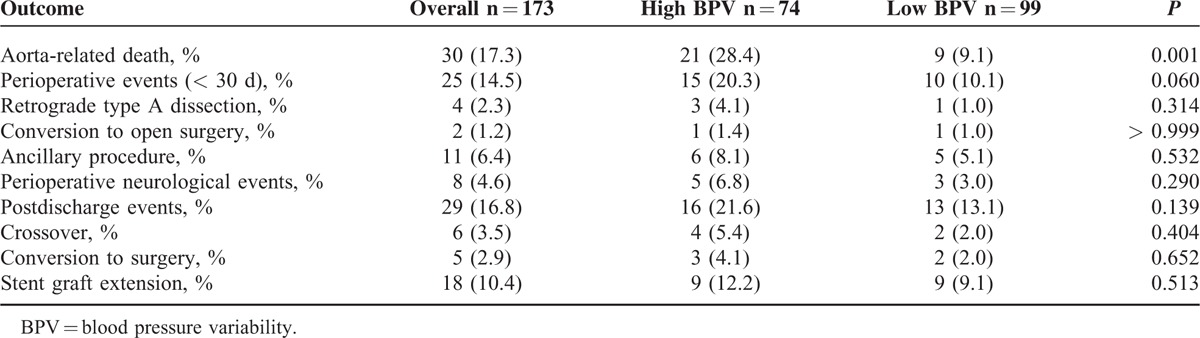
Adverse Events in High and Low BPV Groups

The study cohort was divided into 2 subgroups according to the existence of refractory hypertension or not. In the refractory hypertension group, the risks of aorta-related death and perioperative adverse events in the high BPV subgroup were pronounced higher compared with the low BPV subgroup (41.4% vs 14.3% and 37.9% vs 3.6%, *P* = 0.023 and 0.001, respectively). Meanwhile, the risk of aorta-related death was significantly higher in high BPV without refractory hypertension subgroup (20.0% vs 7.0%, *P* = 0.037) (Table 4).

**TABLE 4:**
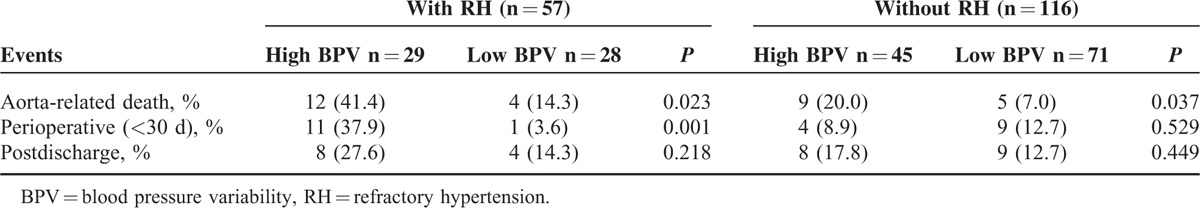
Subgroup Analysis of Adverse Events

### Risk Factors of Mortality

To verify whether BPV was an independent risk factor of aorta-related death, multivariable logistic regression was performed to examine the possible relationship between high BPV and mortality. The model suggested that high BPV was an independent predictor of mortality in our study (95% CI: 1.671–9.587, *P* = 0.002) (Table 5).

**TABLE 5:**
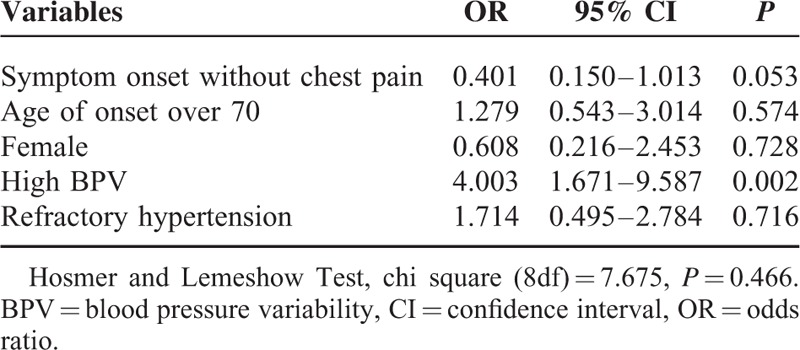
Risk Factors of Aorta-Related Death After Endovascular Therapy for ABADs

### Morphological Aortic Remodeling

In high and low BPV groups, the average aortic maximum diameter before TEVAR were 35.2 ± 5.2 mm and 34.8 ± 5.1 mm, respectively, which decreased to 33.6 ± 4.9 mm (*P* = 0.029) and 33.0 ± 4.8 mm (*P* = 0.007) at 3-month, and 31.7 ± 4.8 mm (*P* < 0.001) and 31.4 ± 4.7 mm (*P* < 0.001) at 6-month follow-up. The aortic maximum diameter decrease ratios in high and low BPV groups were 5.0 ± 2.6% and 5.2 ± 1.9% (*P* = 0.287) at 3-month, and 10.3 ± 3.6% and 9.8 ± 1.8% (*P* = 0.915) at 6-month follow-up, respectively (Figure 2C).

The thrombosis ratios of false lumen in high and low BPV groups before intervention were 8.0 ± 3.4% and 7.1 ± 2.7%, respectively, which increased to 51.8 ± 11.6% (*P* < 0.001) and 72.4 ± 17.5% (*P* < 0.001) at 3-month, and 69.7 ± 7.9% (*P* < 0.001) and 86.4 ± 9.1% (*P* < 0.001) at 6-month follow-up. Moreover, the thrombosis ratio of false lumen in the low BPV group was significantly higher than high BPV group at 3- and 6-month follow-up point (*P* < 0.001, for both) (Figure 2D).

## DISCUSSION

Many studies have concluded that BPV plays an important role in the progress of target organ damage^23,24^ and in triggering cardiovascular events such as stroke,^18,25,26^ myocardial infarction,^27,28^ brain infarction,^29^ and death.^25^ However, the potential prognostic impact of BPV on cardiovascular events is still unclear.^30^ We proposed for the first time that increased BPV was an independent predictor of outcomes after ABAD patients underwent TEVAR.

There was no consensus on blood pressure measurement, which was recommended to be assessed by 24-h ambulatory blood pressure monitoring (measured every 15–30 min) or self-measurement at home.^31^ Variability has mainly been studied during periods of hours on ambulatory monitoring and could also be measured over minutes during a clinic visit, or over days, weeks, and months with home measurements or repeated clinic visits.^32^ Awareness of the sensitivity and emergency of aortic dissection, frequent measurements might make the patients under the risk of stress and result in serious adverse events such as aortic rupture. In the present study, blood pressure was automatically measured and recorded every 4-h using cardiogram monitor during hospitalization. The 4-h interval would not only benefit to acquire continuous information about BPV and avoid frequent disturbance to patients, but also provide close surveillance of the patients. We measured the BPV level and basic characteristics in ABAD patients, and found that there was no significant difference between preoperative and postoperative BPV, which suggested that BPV could be a potential and stable predictor of outcomes in ABAD patients.

Focusing on the potential prognostic significance of BPV after TEVAR, we found the risk of aorta-related death was pronounced higher in the high BPV group. High blood pressure was deemed as a pivotal factor for the development of aortic dissection as well as a main monitoring indicator after definite diagnosis. The underlying principle of aortic dissection treatment was directed at limiting propagation of dissected wall components by control of blood pressure and reduction in pressure development.^11^ The target of lowering systolic blood pressure to 100 to 120 mm Hg was recommended by the European Society of Cardiology in the clinical work.^33,34^ Furthermore, subgroup analysis was performed according to the patients with or without refractory hypertension. We found the aorta-related death was significantly higher in high BPV subgroup regardless of the refractory hypertension. The difference of perioperative events was obvious in high BPV with refractory hypertension subgroup. The results suggested that the effect of BPV was independent of blood pressure level.

Aortic remodeling after TEVAR, described as the true lumen expansion and false lumen obliteration induced by successful coverage of the proximal entry tear,^35^ has been reported as a significant prognostic factor for better long-term results for type B aortic dissection.^36^ Thus, any risk factor which influences aortic remodeling would affect the prognosis of ABAD. We also found the thrombosis ratio of false lumen in the high BPV group was significantly lower than the low BPV group during the follow-up. The result suggested that high BPV might be associated with initial thrombosis process of false lumen, which was a main risk factor for mortality of aortic dissection.^37^ Moreover; hemodynamic change resulted from high BPV might influence the interaction between the stent graft and aortic wall, and therefore lead to stent-related complications such as migration and endoleak.

Blood pressure control is the most important aspect of medical management for aortic dissection. It is necessary and important to recognize the prognostic effect of BPV and supervise it consecutively. According to the current management strategies for ABAD, beta-adrenergic blockers were recommended as the first-line antihypertensive agents to lower the pulse pressure and maintain sufficient end-organ perfusion, whereas vasodilators and calcium channel blockers were applied just as it needed.^11,38,39^ But recent studies showed that beta-adrenergic blockers would increase BPV in blood pressure, and calcium channel blockers could reduce BPV.^40,41^ In fact, the ideal blood pressure-lowering drugs should be able to reduce the blood pressure level as well as maintain hemodynamic stability to promote aortic stability and prevent aortic expansion.^42^ Therefore, stabilization of blood pressure is a potential important target for drug development. In the future, safety testing of all drugs would include assessment of the effects on BPV as well as on mean blood pressure. New drugs or combinations of drugs that yield greater reductions of BPV could have a major beneficial impact on prognosis of TEVAR.^19^

The newest European Society of Cardiology guidelines suggested that the patients with uncomplicated type B aortic dissection can be safely stabilized under medical therapy alone to control pain and blood pressure.^33^ However, the INSTEAD-XL trial recently showed that aorta-specific mortality (19.3% vs 6.9%, *P* = 0.04) and disease progression (46.1% vs 27.0%, *P* = 0.04) were significantly higher in the optimal medical treatment group compared with TEVAR plus medical therapy group after 5 years. Nienaber et al considered that the differences were associated with false lumen thrombosis.^17^ Combination with our results, more attentions should be focused on BPV control for better long-term prognosis in patients with uncomplicated type B aortic dissection.

## LIMITATIONS

This study has several limitations. It was a single-center, retrospective study. Owing to the retrospective nature, evaluation of outcomes of ABAD patients after TEVAR is limited to the results. Computed tomography angiography images were analyzed only 6-month postoperatively. Nevertheless, our study is the first to provide a new insight into the prognostic effect of BPV on aortic dissection, which would be helpful to guide readers to further understanding the clinical problem.

## CONCLUSIONS

Our study proposed that high BPV, which affected the false lumen thrombosis, was an independent risk factor for the prognosis of ABAD. Further studies on BPV might provide new preventive and therapeutic strategies for aortic dissection.
